# Association between Irisin, hs-CRP, and Metabolic Status in Children and Adolescents with Type 2 Diabetes Mellitus

**DOI:** 10.1155/2019/6737318

**Published:** 2019-03-20

**Authors:** Leticia Elizondo-Montemayor, Adrian M. Gonzalez-Gil, Oscar Tamez-Rivera, Carla Toledo-Salinas, Mariana Peschard-Franco, Nora A. Rodríguez-Gutiérrez, Christian Silva-Platas, Gerardo Garcia-Rivas

**Affiliations:** ^1^Tecnologico de Monterrey, Escuela de Medicina y Ciencias de la Salud, Ave. Morones Prieto 3000, Monterrey, N.L. 64710, Mexico; ^2^Tecnologico de Monterrey, Center for Research in Clinical Nutrition and Obesity, Ave. Morones Prieto 300, Monterrey, N.L. 64710, Mexico; ^3^Tecnologico de Monterrey, Cardiovascular and Metabolomics Research Group, Hospital Zambrano Hellion, San Pedro Garza Garcia, P.C. 66278, Mexico; ^4^Hospital Regional de Alta Especialidad Materno Infantil, Ave. San Rafael 450, Guadalupe, P.C. 67140, Mexico

## Abstract

Proinflammatory cytokines and the novel myokine irisin, a cleavage product of FNDC5, have been found to play a role in obesity and type 2 diabetes mellitus (T2DM). Irisin has been shown to increase browning of adipose tissue, thermogenesis, energy expenditure, and insulin sensitivity, yet its association with inflammatory markers is still limited. Circulating irisin has been found to be increased in obesity, while in adult subjects with T2DM decreased levels have been found. However, data establishing the association of circulating irisin in children and adolescents with T2DM has not been described in the literature. The objective of this study was to determine irisin plasma concentration and its association with metabolic and adiposity markers and with hs-CRP, a surrogate marker of inflammation used in clinical practice, in a pediatric population with T2DM. A cross-sample of 40 Mexican children and adolescents aged 7-17 were recruited, 20 diagnosed with T2DM and 20 healthy controls. Plasma irisin levels were found to be lower in the T2DM group compared with controls, which could be attributed to a reduced PGC-1*α* activity in muscle tissue with a consequent decrease in FNDC5 and irisin expression. Irisin concentration was found to be positively correlated with HDL-c, LDL-c, and total cholesterol, while negatively correlated with BMI, waist circumference, and triglycerides. However, after multiple regression analysis, only HDL-c correlation remained significant. hs-CRP was higher in the T2DM group and positively associated with adiposity markers, unfavorable lipid profile, insulin levels, and HOMA-IR, but no association with irisin was found. Given the favorable metabolic effects attributed to irisin, the low plasma levels found in children and adolescents with T2DM could exacerbate the inflammatory and metabolic imbalances and the intrinsic cardiovascular risk of this disease. We propose an “irisin-proinflammatory/anti-inflammatory axis” to explain the role of irisin as a metabolic regulator in obesity and T2DM.

## 1. Introduction

Overweight and obesity are well established risk factors for the development of hypertension, atherogenic dyslipidemia, insulin resistance, and glucose intolerance, all of which carry an increased risk of developing cardiovascular disease and type 2 diabetes mellitus (T2DM) [1, 2] with subsequent increased morbidity and premature mortality. Over the last three decades, overweight and obesity in children and adolescents have become increasingly prevalent worldwide [3]. Both Mexico and the United States present the highest obesity rates in the world [4]. According to the national survey ENSANUT 2016, 34% of children and 36% of adolescents in Mexico were identified as either overweight or obese [5]. While in the United States the prevalence of T2DM in children and adolescents is 4% [6], no data is available for Mexican children. However, approximately half of all pediatric diabetes mellitus cases are estimated to be T2DM [7]. Obesity is considered as a state of systemic low-grade subclinical inflammation, which results in insulin resistance, metabolic abnormalities, and eventually T2DM [8]. Indeed, increased markers of inflammation have been found in serum of obese adults [9–11], but accumulating evidence supports the hypothesis that the obesity-related proinflammatory state begins at and is perpetuated in early childhood [12]. During the last decade, research has also focused on circulating factors involved in the metabolic and inflammatory derangements observed not only in obesity but in T2DM also including inflammatory markers [13–16] and novel myokines, such as irisin, among others [17]. Furthermore, there is overwhelming evidence that T2DM also has an important inflammatory component in its pathogenesis [18]. Some researchers have found increased inflammatory markers in the serum of T2DM children [19] and adolescents [20], which suggests that this inflammatory phenomenon occurs at all ages and justifies further investigation in the less studied pediatric population.

Irisin, a novel myokine discovered in 2012 by Boström and colleagues [21], is a 112-amino acid cleavage product of fibronectin type III domain-containing protein 5 (FNDC5), whose expression is induced by peroxisome proliferator-activated receptor gamma (PPAR-*γ*) and its coactivator peroxisome proliferator-activated receptor-*γ* coactivator 1*α* (PGC1*α*). Irisin was shown to be released by muscle during exercise and to upregulate genes involved in thermogenesis and browning of white adipose tissue (WAT), such as uncoupling protein 1 (UCP-1), which resulted in increased energy expenditure and weight loss in mice fed with a high-fat diet [21]. It was also demonstrated to substantially decrease serum levels of glucose and insulin, thus indicating an improvement in insulin resistance [21]. It was later shown that irisin can also be secreted by subcutaneous adipose tissue [22], being therefore considered an adipomyokine.

Irisin has been proposed to play a role in the pathophysiology of obesity and the consequent metabolic diseases [23, 24]. Most studies have reported a positive correlation between irisin concentration and adiposity and biochemical markers of obesity in adult populations [25–29]. However, the information in children is scarce and rather confusing showing strong contradictory findings [30]. Some studies have shown a positive correlation between irisin and anthropometric and cardiovascular disease markers in obese children [31–34], while others have found an inverse association [35]. Concerning T2DM, 2 meta-analyses have determined that irisin levels are lower in adult patients with T2DM [36, 37], but data establishing the association of irisin concentration in the pediatric population with T2DM has not been described in the literature [19]. Furthermore, there is no available data regarding the association of irisin with an important inflammatory marker in clinical practice such as high-sensitivity C-reactive protein (hs-CRP) in the pediatric population with T2DM. Although hs-CRP has been used clinically as an inflammatory marker in this population [38], its relationship with irisin concentration has not been studied. Therefore, the objective of this study was to investigate irisin concentration and its association with hs-CRP, as well with metabolic and anthropometric parameters in children and adolescents with T2DM compared with healthy controls.

## 2. Materials and Methods

### 2.1. Population

A cohort of 40 Mexican children and adolescents aged 7-17 (20 boys and 20 girls) were recruited, 20 children and adolescents diagnosed with T2DM and 20 healthy controls. Inclusion criteria for the T2DM group included to have been previously diagnosed with T2DM according to the American Diabetes Association (ADA) criteria. Children were diagnosed by fasting plasma glucose ≥ 126 mg/dL (7.0 mmol/L) [39]. Healthy controls were required to have normal weight according to the Centers for Disease Control and Prevention (CDC) criteria (body mass index percentile (BMIp) ≥5th and <85th) [40], no metabolic abnormalities on blood samples, and no previously diagnosed cardiometabolic diseases, as well no antihypertensive, lipid, or sugar lowering medications use. Written informed consent was obtained from legal guardians prior to the inclusion in this study. Approval by the Ethics and Research Committees of the School of Medicine, Tecnologico de Monterrey was obtained.

### 2.2. Vital Signs and Anthropometric Parameters

Anthropometric variables and blood pressure were measured by qualified medical personnel. Blood pressure measurements were obtained by triplicate with a mercury sphygmomanometer, using an appropriate cuff size and with the patient in sitting position. Anthropometric parameters were obtained according to standardized protocols [41]. Weight in kilograms (kg) was rounded to the nearest decimal point and measured with an age-appropriate scale (TANITA® BF-689; TANITA Corporation of America Inc., Arlington Heights, Illinois, USA); height in centimeters (cm) was measured with a stadiometer (SECA® 217, SECA Mexico, Mexico City, Mexico); body mass index (BMI) was calculated as kg/m^2^; waist circumference (WC) and hip circumference (HC) in cm were obtained with a standard fiber optic measuring tape.

### 2.3. Laboratory Studies

Blood samples were taken from each subject by peripheral venipuncture after an overnight 12-hour fast. Samples were then centrifuged to obtain plasma and serum and were then frozen at −80°C for further processing. Fasting serum glucose levels were measured by the hexokinase (HK)/glucose-6-phosphate dehydrogenase (G-6-PDH) method, using the Glucose 3L82 (304772/R02; DENKA SEIKEN Co. Ltd., Tokyo, Japan) reagent kit on the Architect *c*Systems™. Serum insulin concentrations were obtained by chemiluminescent microparticle immunoassay, using the ARCHITECT Insulin Reagent kit 8K41-27 (G6-2892/R03; Abbot Laboratories Diagnostic Division, IL, USA). Total cholesterol was measured using the Cholesterol Reagent kit 7D62 (304796/R02; Abbot Laboratories Diagnostic Division, IL, USA) on the Architect *c*Systems™ through enzymatic methodology. High-density lipoprotein cholesterol concentrations were measured by the accelerator selective detergent method using the Ultra HDL 3K33-21 assay (306571/R03; Abbot Laboratories Diagnostic Division, IL, USA). Quantitation of triglycerides in plasma was obtained through the glycerol-phosphate-oxidase reaction, using the Triglyceride 7D74-20 (30-3140/R3; Abbot Laboratories Diagnostic Division, IL, USA) reagent kit on the Architect *c*Systems™ and the AEROSET system. High-sensitivity C-reactive protein levels were determined by quantitative immunoturbidimetric methodology, using the CRP Vario 6k26-30 and 6k26-41 kits (306731/R04; Abbot Laboratories Diagnostic Division, IL, USA) on the Architect *c*Systems™. Irisin was measured by sandwich enzyme-linked immunosorbent assay (ELISA) method with an irisin (human) ELISA kit (SK00170-08) following the manufacturer's instructions (Avisera Bioscience Inc., Santa Clara, California, USA). The sensitivity of this assay is 0.1 ng/mL; its standard curve linear range is 0.8-51.2 ng/mL, and intra- and interassay variations are 4-6% and 8-10%, respectively. For cytokine measurement, human TNF-*α* (catalogue number 430306) and human IL-6 ELISA MAX (catalogue number 430503) sets, both from BioLegend (San Diego, CA, USA), were used following the manufacturer's instructions. Sensitivity for the assay is 2 pg/mL.

### 2.4. Statistical Analyses

Statistical analyses were performed using Microsoft Excel® (version 16.17, Microsoft Corporation, Redmont, Washington, USA), IBM SPSS® (version 21.0; SPSS Inc., Armonk, New York, USA), and GraphPad Prism (version 6.0, GraphPad Software, La Jolla California, USA). D'Agostino-Pearson tests were used to evaluate normality of the sample distribution. Unpaired *t*-tests and Mann-Whitney tests were conducted for parametric and nonparametric data, respectively, to compare anthropometric and metabolic variables between the two groups. Chi-square and Fisher's exact tests, when deemed appropriate, were conducted to evaluate differences in proportions of clinical categorical variables between groups. Spearman's rank correlation test was used to assess the relation between levels of irisin and all clinical and biochemical parameters. Stepwise multiple linear regression analysis was also performed to adjust for gender, age, and BMI. A *p* value of <0.05 was considered statistically significant for all analyses.

## 3. Results

### 3.1. Demographic and Clinical Parameters

Table 1 shows the demographic and clinical characteristics of the pediatric population classified into two groups: T2DM (*n* = 20) and healthy controls (*n* = 20) with an equal distribution of male and female participants. The subject's mean age was 12.58 (±2.47) years. Ninety percent of the T2DM group were found to be either obese (70%) or overweight (20%), while none of the healthy control group subjects were. The mean age of the T2DM children was significantly greater (13.9 (11-17)) compared with that of the control group (11.2 (7-16)) (*p* = 0.0002). Acanthosis nigricans was found in ninety-five percent of the T2DM patients, but in none of the subjects from the control group. All T2DM subjects presented Tanner developmental stages 3-5, while controls showed equal distribution between Tanner 1-2 and 3-5 stages.

### 3.2. Anthropometric and Metabolic Parameters

Table 2 describes the anthropometric, metabolic, and clinical variables for the T2DM and healthy control groups. Body weight (kg) (68.40), height (cm) (1.59), BMI (27.8), and BMI percentiles (96.4) were significantly higher in the T2DM group compared with the control group (34.90, 1.45, 17.70, and 54.15), respectively. As well, waist circumference (96.10), hip circumference (102.2), and waist-height ratio (0.596) were also significantly higher in the T2DM group compared with the healthy group (63.60, 65.40, and 0.439), respectively.

Regarding metabolic and clinical variables, fasting glucose (114.00 (92.00-158.00)), triglycerides (157.00 (135.20-199.50)), insulin (23.3 (10.63-30.93)), HOMA-IR (8.35 (2.85-12.78)), and systolic blood pressure (112.00 (104.30-120.00)) were significantly higher and HDL-c (37.50 (31.25-44.75)) significantly lower in the T2DM group compared with the control group. As well, hs-CRP was significantly higher in T2DM subjects (1.32 ± 0.62 (1.03-1.61)) compared with the control subjects (0.83 ± 0.39 (0.64-1.02)) (*p* = 0.006). The normal insulin range (mIU/L) reported by the laboratory department is 5.0-20.0 (mIU/L).

### 3.3. Irisin Plasma Levels

**Figure 1:**
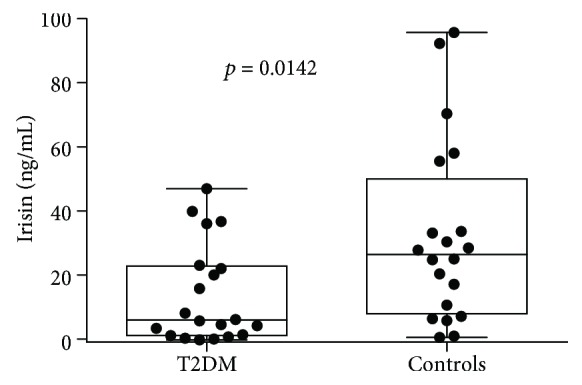
Irisin levels in studied groups. T2DM = type 2 diabetes mellitus.

Figure 1 shows irisin plasma levels in the T2DM group compared with the control group. Irisin concentration was significantly lower for the T2DM group (6.84 (2.07-23.72)) compared with the healthy control group (27.35 (8.91-50.95)) (*p* = 0.014).

### 3.4. Correlation between Plasma Irisin Levels and hs-CRP, Anthropometric, Metabolic, Clinical, and Inflammatory Parameters

**Figure 2:**
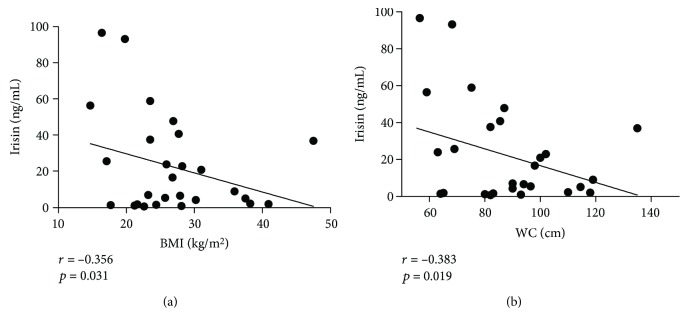
Correlation between irisin plasma levels and anthropometric parameters. Association of plasma irisin levels with BMI (a) and waist circumference (b). BMI = body mass index; WC = waist circumference.

**Figure 3:**
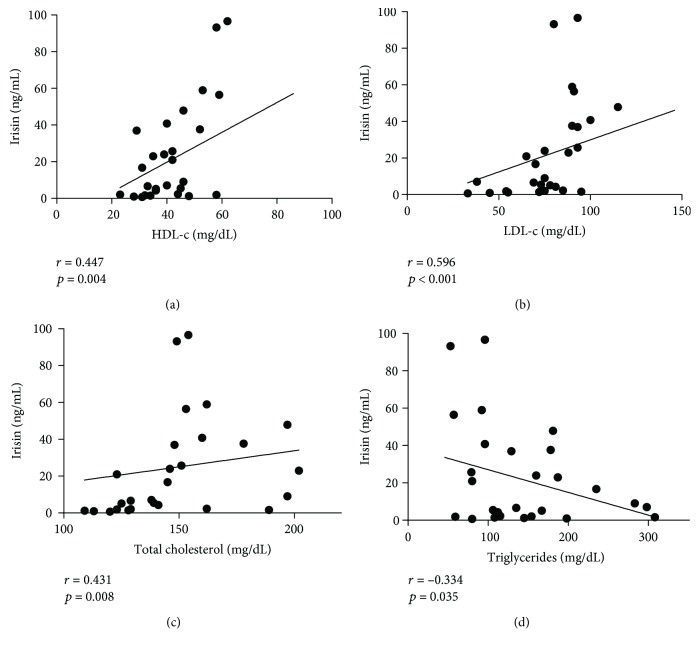
Irisin plasma level correlations with metabolic parameters. Association of plasma irisin levels with high-density lipoprotein cholesterol (a), low-density lipoprotein cholesterol (b), total cholesterol (c), and triglycerides (d).

Table 3 shows Spearman's correlation coefficients between irisin concentration and the anthropometric and metabolic parameters. Irisin levels were found to be inversely correlated with height (*r* = −0.380), weight (*r* = −0.356), BMI (*r* = −0.356), WC (*r* = −0.383), and HC (*r* = −0.391) (*p* < 0.05). Regarding metabolic and clinical parameters, plasma irisin levels were found to be positively correlated with total cholesterol (*r* = 0.431), LDL-c (*r* = 0.596), HDL-c (*r* = 0.447) (*p* < 0.01), and DBP percentile (*r* = 0.350, *p* < 0.05). However, after multiple linear regression analysis adjusting for age, gender, and BMI, only HDL-c showed a positive correlation with irisin (*B* = 0.813, *p* = 0.009). Scatter plots for anthropometric and metabolic data are shown in Figures 2 and 3, respectively. Of note, subjects with acanthosis showed significantly greater insulin levels (23.7 (10.2-31.3)) than those without acanthosis (7.0 (5.05-8.20)) (*p* < 0.001), alike for HOMA-IR (8.00 (3.80-12.9) and 1.20 (1.00-1.75), respectively.

### 3.5. Correlation between hs-CRP, Irisin, Anthropometric, and Metabolic Parameters

hs-CRP was found to be significantly associated with anthropometric markers of obesity, such as weight (*r* = 0.509), BMI (*r* = 0.435), BMI% (*r* = 0.518), WC% (*r* = 0.542), W/ht ratio (*r* = 0.463) (*p* < 0.01), and WC (*r* = 0.415, *p* < 0.05). hs-CRP was also found to be positively correlated with metabolic markers such as triglycerides (*r* = 0.374, *p* < 0.05) and negatively correlated with HDL-c (*r* = −0.327, *p* < 0.05). Interestingly, we found hs-CRP to be positively associated with serum insulin levels (*r* = 0.394, *p* < 0.05) and with HOMA-IR (*r* = 0.393, *p* < 0.05). No association was found between hs-CRP and irisin levels.

## 4. Discussion

### 4.1. Irisin Levels in Children and Adolescents with T2DM

Irisin was initially described as a myokine in transgenic mice overexpressing *Ppargc1a*, a gene that encodes the transcription cofactor peroxisome proliferator-activated receptor-*γ* coactivator 1*α* (PGC1*α*) [21], a protein whose expression has been found to be increased by exercise. Irisin has been described to be involved in the regulation of mitochondrial biogenesis and function in muscle cells, FNDC5 expression, and other metabolic pathways [23]. Irisin has also been linked to an array of clinical entities, especially to metabolic diseases including obesity, T2DM, and cardiovascular risk factors [24].

To our knowledge, this is the first study to evaluate irisin levels in the pediatric population with T2DM. We found irisin levels to be decreased in children and adolescents with T2DM compared with control subjects. Similar results have been shown in adults with T2DM. [42–45]. The higher irisin levels found in our normal weight group support the original role described for irisin related to an increased thermogenesis and energy expenditure mediated through UCP1 [21], leading to browning of adipose tissue. *In vitro* and animal studies have shown irisin to have both direct and indirect effects on metabolic pathways, acting mainly on adipose tissue, muscle, and the liver. [23]. To begin with, in adipose tissue, irisin has been able to enhance glucose uptake [46], to stimulate lipolysis, and to inhibit lipid accumulation [47]. In addition, in muscle, irisin appears to modulate metabolic processes by activating AMP-activated protein kinase (AMPK), thereby increasing glucose uptake, lipid uptake, and metabolism and decreasing glycogenolysis and gluconeogenesis [48]. Finally, in hepatocytes, irisin has been shown to reduce oxidative stress [49, 50], to promote glycogenesis, to inhibit gluconeogenesis [51], and to reduce lipogenesis and lipid accumulation [49]. Overall, considering these favorable metabolic effects, the decreased levels of irisin observed in our T2DM pediatric population could potentially exacerbate the decreased glucose uptake in muscle and other metabolic derangements observed in peripheral tissues of T2DM subjects. The low irisin levels could also alter lipid uptake and metabolism, promoting lipogenesis and lipid accumulation, which may well increase the cardiovascular risk in children and adolescents with T2DM.

Although the mechanisms underlying the lower levels of irisin observed in T2DM subjects are not clearly understood, some hypotheses have been described. Lower irisin concentration in T2DM patients could be explained by a reduced PGC-1*α* activity in muscle tissue of T2DM individuals, which was observed long before the discovery of irisin [52]. Lower levels of circulating irisin have been found in a sample of 96 Asian adults with T2DM compared with 60 nondiabetic controls [44]. Consequently, the reduction in PGC-1*α* activity in muscle tissue of T2DM patients would lead to a resultant decrease in FNDC5 and irisin expression. As well, insulin resistance, which ultimately results in hyperglycemia and increased circulating free fatty acids [53], has also been proposed as responsible for the decrease in PGC-1*α* activity [54]. In addition, free fatty acids and glucose, in turn, could be the direct mediators of decreased irisin expression, as indicated by a study conducted by Kurdiova and colleagues in a cohort of 99 European sedentary adults, divided into lean (*n* = 29), obese (*n* = 29), prediabetic (*n* = 25), and T2DM (*n* = 16) subjects. Hyperglycemia and triglyceridemia were found to be negatively associated with adipose tissue FNDC5 mRNA expression and with circulating irisin. This finding was further reinforced by the observation that FNDC5 mRNA expression in muscle cells obtained from these subjects was found to be lower after an *in vitro* treatment with palmitate (a saturated fatty acid) and with glucose [42]. Therefore, since there is no previous data associating irisin plasma levels in T2DM in children and adolescents and considering the findings in the adult population, the lower irisin levels found in our cohort could be attributed to a reduced PGC-1*α* activity in muscle tissue with a consequent decrease in FNDC5 and irisin expression. Furthermore, the hyperglycemia and hypertriglyceridemia found in our T2DM group might also be negatively associated with adipose tissue FNDC5 mRNA expression and circulating irisin.

Patients with T2DM have metabolic imbalances in which irisin may play a role [24]. Relationships between irisin concentration and specific common metabolic parameters in the clinical setting have been scarcely studied in children resulting in contradictory findings [30]. Our results show irisin concentration to be positively correlated with HDL-c, total cholesterol, and LDL-c levels, and negatively correlated with TG. However, after multiple linear regression analysis, only HDL-c correlation remained significant, which might be attributed to the higher levels shown by the normal weight group compared with the T2DM group. Since HDL-c has been found to have a protective anti-inflammatory role against cardiovascular disease and has been associated with preserved endothelial function [55], higher irisin levels found in the control group may favor the maintenance of vascular function in normal weight children. On the other hand, endothelial function has been shown to be impaired in adolescents with T2DM [56]. Interestingly, a positive correlation between irisin and endothelial progenitor cells (EPCs) has been found in a cohort of 24 nondiabetic overweight and obese children. The authors hypothesized that irisin could represent a compensatory protective effect against endothelial damage in obesity through EPC activation and/or mobilization [57]. Given that lower irisin levels were found in our cohort of T2DM children and adolescents, the compensatory role of irisin on vascular damage through the EPC pathway may be lost, further increasing their cardiovascular risk.

### 4.2. Irisin and Inflammation in Children and Adolescents with Obesity and T2DM

One of the features that characterizes most of the population with T2DM is central obesity. Regarding anthropometric markers of adiposity, most studies in nondiabetic obese adults have shown increased circulating levels of irisin [24] and a positive association with BMI, WC, and body fat percentage [28, 29, 58–60], but in the pediatric population findings are still controversial and attributed to differences in body composition and its variations during growth and development [30]. In our study, we found irisin levels to be negatively correlated with BMI and WC. In agreement with our findings, Shim et al. found irisin plasma levels to be higher in normal weight children compared with nondiabetic overweight/obese subjects [35], while some authors have found an increased irisin concentration and a positive correlation with BMI, WC, fat mass, and % body fat [31–33, 61, 62] in obese children and still others have found no correlation at all [63]. The increase in irisin levels observed in obesity may simply be the result of an excessive adipose tissue as an important source of irisin, which may increase insulin sensitivity and energy expenditure as a compensatory mechanism to counteract the deleterious metabolic effects of excess adiposity, namely, insulin and catecholamine resistance [23, 64]. On the other hand, the increased circulating irisin may represent an “irisin resistance” state, similar to the insulin and leptin resistance that results in hyperinsulinemia and hyperleptinemia observed in obese individuals [24]. Considering that virtually all overweight and obese subjects in our study were diabetic, while none of the children in the control group were, the diabetic condition itself could be accountable for the inverse relationship between irisin levels and BMI and WC.

Obesity has long been considered as a state of low-grade chronic inflammation [65]. Recent evidence has shown that this inflammatory state begins and is maintained early in childhood [12], but evidence of this association in children with T2DM is limited. Although the precise stimuli that promote obesity-associated inflammation are poorly understood, it has been hypothesized that hypertrophic adipose tissue induces adipocyte apoptosis, hypoxia, and mechanical stress that activate proinflammatory pathways, with a resultant increase in proinflammatory cytokine and chemokine expression in adipocytes, which results in recruitment of macrophages to adipose tissue and their polarization to the M1 proinflammatory phenotype [64]. Furthermore, the increased circulating levels of free fatty acids associated with obesity may also directly initiate an inflammatory reaction [13, 66]. In turn, the inflammatory response induces insulin resistance through several mechanisms [67]. For instance, proinflammatory cytokines such as TNF-*α*, secreted locally by adipose tissue macrophages, have been found to be likely mediators of insulin resistance [64, 67]. High-sensitivity C-reactive protein (hs-CRP), synthesized in the liver in response to circulating proinflammatory cytokines [68], has been extensively used as a surrogate marker of nonspecific inflammation in a variety of clinical settings [69] in which evaluation of the cytokine profile is not always available and is rather expensive. Noteworthy, evidence of the association of a proinflammatory profile in children and adolescents with T2DM is scarce. The only available evidence to date has shown higher concentrations of TNF-*α*, IL-1*β*, and hs-CRP in obese Caucasian adolescents with T2DM compared with obese controls without T2DM [20]. In addition, high levels of C-reactive protein (CRP) and IL-6 have been found to predict the onset of T2DM [15]. As expected, our results show hs-CRP levels to be higher in the T2DM group compared with the normal weight group. hs-CRP levels were also found to be positively associated with WC, W/ht ratio, and triglycerides, while a negative association with HDL-c was found, all markers of central adiposity and the metabolic imbalance characteristic of both obesity and T2DM. Finally, hs-CRP was found to be positively associated with insulin levels and HOMA-IR, the hallmark of T2DM. Thus, the increased inflammatory response found in the T2DM children might be attributed to the hypertrophic adipose tissue and increased circulating free fatty acids, among other stimuli.

An interesting possibility of an “irisin-inflammatory/anti-inflammatory axis” could arise, supported by evidence related to obesity and diabetes [68]. At the anti-inflammatory side of the axis, after treatment with irisin, a decrease in the expression of TNF-*α*, IL-1*β*, IL-6, and MCP-1 by LPS-activated murine macrophages [70] and by adipocytes [71] has been demonstrated. Similarly, decreased M1 polarization and proinflammatory cytokine expression after treating murine macrophages with irisin's precursor, FNDC5, has been reported [72]. As well, irisin has been shown to promote polarization of adipose tissue macrophages to the alternative M2 anti-inflammatory phenotype [73]. In studies in humans, mRNA expression of FNDC5 was found to be negatively associated with TNF-*α* expression in visceral adipose tissue, while positively associated with the expression of IL-10, an anti-inflammatory cytokine, in subcutaneous adipose tissue. These findings were reported in a cohort of obese and normal weight Caucasian individuals with and without T2DM [43], a finding that could represent the interaction of irisin with adipose tissue macrophages. At the proinflammatory side of this axis, TNF-*α* and IL-1*β* have been shown to inhibit FNDC5 expression in murine myotubes [74], possibly decreasing irisin secretion. A positive association between irisin and hs-CRP might suggest that irisin is secreted as a counterregulatory mechanism against inflammation, while a negative association could indicate that irisin expression is being suppressed by ongoing inflammation. However, we did not find any association between hs-CRP and plasma irisin levels, which could be attributed among other factors, to our small sample size. To our knowledge, the association between irisin and inflammatory markers in the pediatric population is greatly limited. Viitasalo et al. [75] found irisin to be positively associated with TNF-*α* and IL-6 in nondiabetic Caucasian children aged 6-8 years. The authors attributed the observed positive correlation between irisin and proinflammatory cytokines to a compensatory increase of irisin to limit lipid accumulation and inflammatory changes in the liver of children at increased risk of NAFLD. In another study conducted by Sarac et al., higher levels of irisin as well as of hs-CRP were found in children with acute appendicitis compared with controls [76]. This finding might suggest that irisin secretion increases in conditions characterized by a state of inflammation.

### 4.3. The Role of Irisin in the Inflammatory State and Metabolic Imbalances of T2DM

**Figure 4:**
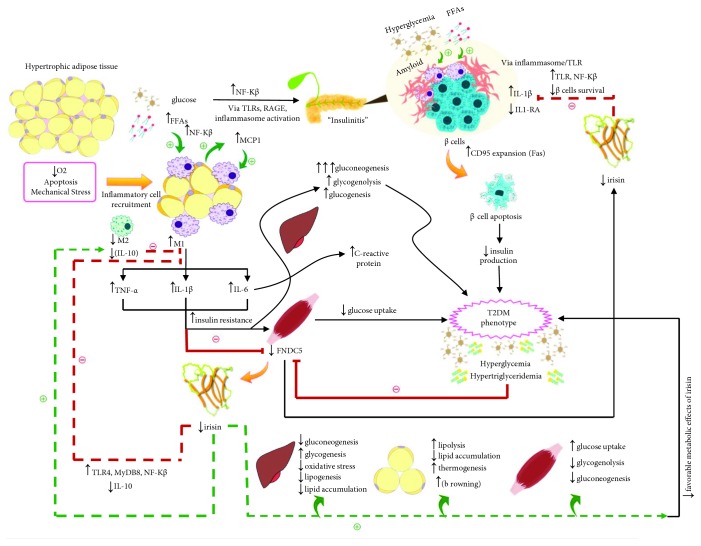
Possible irisin-inflammatory cross talk in overt T2DM and exacerbation of metabolic derangements due to hypoirisinemia. Dotted lines indicate the theoretical compensatory effects of higher irisin levels in context of obesity, which may be impaired in patients with T2DM who have decreased levels of irisin. Continuous lines indicate an effect that could be observed in context of T2DM. Green lines indicate stimulation and red lines indicate inhibition. FFAs = free fatty acids; FNDC5 = fibonectin type III domain-containing protein 5; IL-1RA = interleukin-1 receptor antagonist; IL-1*β* = interleukin-1*β*; IL-6 = interleukin-6; IL-10 = interleukin-10; LPS = lipopolysaccharide; M1 = classically activated (proinflammatory) macrophage; M2 = alternatively activated (anti-inflammatory) macrophage; MCP1 = monocyte chemoattractant protein 1; MyD88 = myeloid differentiation primary response 88; NF-*κ*B = nuclear factor kappa B; TLR = toll-like receptor; RAGE = receptor for advanced glycation end products; T2DM = type 2 diabetes mellitus; TNF-*α* = tumor necrosis factor-alpha.

Figure 4 shows the hypothesis for our findings correlating the integration between the possible irisin-inflammatory/anti-inflammatory axis, as well as the role of irisin in T2DM. In summary, adipose tissue hypertrophy may lead to hypoxia, adipocyte apoptosis, and mechanical stress, all of which activate an inflammatory response in adipocytes that causes the release of cytokines and chemokines, including MCP-1. This is followed by recruitment and differentiation of monocytes to proinflammatory M1 classically activated macrophages [64]. M1 macrophages secrete the proinflammatory cytokines TNF-*α*, IL-1*β*, and IL-6, which promote insulin resistance and the accompanying deleterious metabolic effects [67], as well as synthesis of C-reactive protein [77]. Irisin expression early in obesity, before development of overt diabetes, may be increased in adipose tissue as a compensatory response to counteract insulin resistance and to increase energy expenditure [24]. As well, increased irisin levels inhibit the secretion of proinflammatory cytokines and promote secretion of the anti-inflammatory cytokine IL-10 by interacting with infiltrating immune cells in adipose tissue and stimulating macrophage polarization from M1 to M2 phenotype [43, 70, 72, 73, 78].

However, at the other end of the spectrum, in obese type 2 diabetic individuals, irisin may be suppressed due to either insulin resistance itself, which could indirectly inhibit the secretion of irisin by glucotoxic or lipotoxic mechanisms [42]. Alternatively, an overwhelming proinflammatory milieu directly inhibits irisin production in muscle [74]. Both may further exacerbate the deleterious inflammatory and metabolic imbalances present in T2DM. Furthermore, decreased irisin levels might exacerbate the pancreatic islet infiltration of immune cells (“insulinitis”) observed in T2DM [18]. This effect might be attributed to a decreased inhibitory effect over the local production of IL-1*β* by interacting with local macrophages [70], which the presence of continued hyperglycemia, elevated free fatty acids, and amyloid deposition [79] leads to continued islet inflammation, *β*-cell apoptosis, and consequent decreased insulin secretion, observed late in the evolution of T2DM [18]. In addition, irisin has been found to be positively associated with circulating insulin and HOMA-*β* even in healthy subjects, indicating that it may play a role in the regulation of *β*-cell function [23, 80].

The study has several limitations. The sample size was small and all subjects are from Hispanic ethnicity; thus, the generalizability of the results is limited. Due to the small sample size and transversal design, direct establishment of a correlation of irisin with inflammatory markers is not possible. However, the study presents several strengths. This is the first study to evaluate irisin concentration in a children and adolescents with T2DM and its association with the metabolic and anthropometric markers in this T2DM population. The role of irisin in inflammation, a well-known element in T2DM patients, has been proposed. In addition, the novel figure highlights the role of irisin in inflammation and as a metabolic regulator in obesity and T2DM based on literature findings.

## 5. Conclusions

This is the first study to demonstrate decreased levels of irisin in children and adolescents with T2DM compared with healthy controls. Based on experimental evidence, decreased circulating irisin in the pediatric population with T2DM could be attributed to decreased muscle secretion of FDNC5. After adjustment for possible confounders, we found a positive correlation between irisin and HDL-c, which could suggest that irisin has a potential protective mechanism against endothelial damage and vascular disease. Importantly, we observed hs-CRP to be higher in the T2DM group and to be positively associated with anthropometric and metabolic markers of T2DM, which indicates that a systemic inflammatory state is present in the pediatric population with T2DM. Irisin lower concentrations in this population could potentially exacerbate the metabolic and inflammatory components of this disease. Larger clinical studies are needed to elucidate the relationship between circulating inflammatory markers and irisin in children with T2DM, as well as experimental studies to elucidate the mechanisms of hypoirisinemia in T2DM, as irisin may ultimately play a role as a therapeutic agent in obesity and in T2DM.

## Figures and Tables

**Table 1:** Demographic and clinical characteristics of the population.

Parameter	T2DM (*n* = 20)	Healthy controls (*n* = 20)	*p* value
Male	10 (50)	10 (50)	1.00
Female	10 (50)	10 (50)	
Age in years	13.9 ± 1.52	11.2 ± 2.67	**<0.001**
Obesity^a^	14 (70)	0 (0)	**<0.001**
Overweight^b^	4 (20)	0 (0)	
Normal weight	2 (10)	20 (100)	
Acanthosis nigricans	19 (95)	0 (0)	**<0.001**
Tanner 1-2	0 (0)	11 (55)	**<0.001**
Tanner 3-5	20 (100)	9 (45)	

T2DM = type 2 diabetes mellitus. ^a^Obesity was defined as BMI ≥ 95th percentile according to CDC criteria. ^b^Overweight was defined as BMI ≥ 85th percentile and <95th percentile according to CDC criteria. Data are presented as absolute number and percentage (%) unless specified otherwise.

**Table 2:** Anthropometric, metabolic, clinical, and inflammatory parameters for the T2DM and healthy control groups.

Parameter	T2DM (*n* = 20)	Healthy controls (*n* = 20)	*p* value
Weight (kg)	68.4 (61.60-90.85)	34.90 (26.50-44.80)	**<0.001**
Height (m)	1.59 (1.55-1.66)	1.45 (1.33-1.60)	**0.002**
BMI (kg/m^2^)	27.8 (24.73-34.68)	17.70 (16.35-18.95)	**<0.001**
BMI%	96.4 (89.73-99.10)	54.15 (25.45-70.20)	**<0.001**
WC (cm)	96.13 ± 16.72	63.6 ± 6.14	**<0.001**
WC%	85.00 (50.00-93.75)	15 (10.00-25-00)	**<0.001**
HC (cm)	102.2 ± 16.89	65.4 ± 6.73	**<0.001**
WHI	0.939 ± 0.07	0.968 ± 0.01	0.081
W/ht	0.596 ± 0.093	0.439 ± 0.016	**<0.001**
SBP (mmHg)	112.00 (104.3-120.00)	98.00 (96.00-104.00)	**<0.001**
SBP%	58.00 (31.2-89.00)	39.00 (26.50-42.00)	**0.027**
DBP (mmHg)	67.50 (62.25-78.75)	64.00 (63.00-67.00)	0.249
DBP%	57.00 (43.25-90.75)	65 (53.00-67.50)	0.970
Glc (mg/dL)	114.5 (92.00-205.30)	84.00 (71.50-88.50)	**<0.001**
Serum insulin (mIU/L)	23.30 (10.63-30.93)	6.60 (4.83-7.98)	**<0.001**
HOMA-IR	8.35 (3.85-12.78)	1.20 (1.00-1.65)	**<0.001**
TC (mg/dL)	149.50 ± 29.24	153.4 ± 22.93	0.658
HDL-c (mg/dL)	37.50 (31.25-44.75)	50.50 (43.25-58.75)	**<0.001**
LDL-c (mg/dL)	74.90 ± 20.63	100.3 ± 24.02	**0.001**
TG (mg/dL)	157.00 (112.8-195.30)	87.50 (70.75-99.50)	**<0.001**
hs-CRP (mg/L)	1.32 ± 0.62	0.83 ± 0.39	**0.006**
TNF-*α* (pg/mL)	2.12 (1.92-2.51)	2.58 (2.25-2.85)	0.167
IL-6 (pg/mL)	3.38 (2.90-3.84)	4.13 (2.95-4.68)	0.104

Values are presented as median and interquartile range for nonparametric data and as mean and standard deviation for parametric data. BMI = body mass index; DBP = diastolic blood pressure; Glc = fasting glucose; HC = hip circumference; HDL-c = high-density lipoprotein cholesterol; hs-CRP = high sensitivity C-reactive protein; LDL = low-density lipoprotein cholesterol; HOMA-IR = homeostatic model assessment of insulin resistance; SBP = systolic blood pressure; T2DM = type 2 diabetes mellitus; TC = total cholesterol; TG = triglycerides; WC = waist circumference; WHI = waist-hip index; W/ht = waist-to-height ratio; % = percentile for age and gender.

**Table 3:** Correlation between irisin concentration and anthropometric, metabolic, and inflammatory parameters.

	Height	Weight	BMI	BMI%	WC	WC%	HC	WHI	W/ht	SBP	SBP%	DBP	DBP%	TC	TG	HDL-c	LDL-c	Glc	Insulin	HOMA	hs-CRP
Irisin	**-0.380** ^∗^	**-0.313** ^∗^	**-0.356** ^∗^	-0.250	**-0.383** ^∗^	-0.208	**-0.391** ^∗^	0.168	-0.268	-0.166	0.051	0.263	**0.350** ^∗^	**0.431** ^∗∗^	**-0.334** ^∗^	**0.447** ^∗∗^	**0.596** ^∗∗^	-0.173	-0.040	-0.052	0.036
Height		**0.786** ^∗∗^	**0.605** ^∗∗^	**0.474** ^∗∗^	**0.711** ^∗∗^	**0.497** ^∗∗^	**0.703** ^∗∗^	-0.183	**0.355** ^∗^	**0.536** ^∗∗^	-0.064	0.092	-0.272	-0.183	0.349^∗^	**-0.374** ^∗^	**-0.499** ^∗∗^	0.254	0.281	0.243	0.141
Weight			**0.956** ^∗∗^	**0.882** ^∗∗^	**0.934** ^∗∗^	**0.808** ^∗∗^	**0.946** ^∗∗^	-0.240	**0.735** ^∗∗^	**0.628** ^∗∗^	0.154	0.118	-0.186	-0.134	**0.523** ^∗∗^	**-0.573** ^∗∗^	**-0.506** ^∗∗^	**0.624** ^∗∗^	**0.575** ^∗∗^	**0.589** ^∗∗^	**0.509** ^∗∗^
BMI				**0.941** ^∗∗^	**0.907** ^∗∗^	**0.794** ^∗∗^	**0.935** ^∗∗^	-0.261	**0.788** ^∗∗^	**0.598** ^∗∗^	0.209	0.099	-0.151	-0.098	**0.563** ^∗∗^	**-0.634** ^∗∗^	**-0.435** ^∗∗^	**0.601** ^∗∗^	**0.656** ^∗∗^	**0.634** ^∗∗^	**0.435** ^∗∗^
BMI%					**0.812** ^∗∗^	**0.836** ^∗∗^	**0.838** ^∗∗^	-0.173	**0.782** ^∗∗^	**0.524** ^∗∗^	0.261	0.056	-0.086	-0.058	**0.588** ^∗∗^	**-0.537** ^∗∗^	**-0.398** ^∗^	**0.659** ^∗∗^	**0.629** ^∗∗^	**0.655** ^∗∗^	**0.518** ^∗∗^
WC						**0.896** ^∗∗^	**0.969** ^∗∗^	-0.140	**0.871** ^∗∗^	**0.720** ^∗∗^	0.322	0.157	-0.121	-0.122	**0.548** ^∗∗^	**-0.612** ^∗∗^	**-0.465** ^∗∗^	**0.555** ^∗∗^	**0.662** ^∗∗^	**0.608** ^∗∗^	**0.415** ^∗^
WC%							**0.861** ^∗∗^	-0.061	**0.952** ^∗∗^	**0.649** ^∗∗^	**0.468** ^∗∗^	0.125	-0.006	-0.085	**0.562** ^∗∗^	**-0.490** ^∗∗^	**-0.376** ^∗^	**0.582** ^∗∗^	**0.635** ^∗∗^	**0.634** ^∗∗^	**0.542** ^∗∗^
HC								**-0.334** ^∗^	**0.846** ^∗∗^	**0.639** ^∗∗^	0.227	0.117	-0.177	-0.151	**0.558** ^∗∗^	**-0.641** ^∗∗^	**-0.478** ^∗∗^	**0.604** ^∗∗^	**0.679** ^∗∗^	**0.653** ^∗∗^	**0.393** ^∗^
WHI									-0.053	0.047	0.298	0.173	0.320	0.200	-0.050	0.181	0.163	-0.198	-0.180	-0.265	0.187
W/Ht										**0.606** ^∗∗^	**0.490** ^∗∗^	0.123	0.009	-0.139	**0.554** ^∗∗^	**-0.547** ^∗∗^	**-0.349** ^∗^	**0.520** ^∗∗^	**0.706** ^∗∗^	**0.656** ^∗∗^	**0.463** ^∗∗^
SBP											**0.735** ^∗∗^	**0.498** ^∗∗^	0.289	-0.061	**0.441** ^∗∗^	**-0.412** ^∗^	-0.295	0.295	**0.388** ^∗^	0.287	0.210
SBP%												**0.531** ^∗∗^	**0.596** ^∗∗^	0.073	0.362^∗^	-0.192	-0.002	0.151	0.252	0.167	0.258
DBP													**0.860** ^∗∗^	**0.366** ^∗^	0.162	0.163	0.171	0.100	0.083	0.026	0.028
DBP%														0.288	0.076	0.263	0.266	0.006	-0.081	-0.109	-0.098
TC															0.099	**0.377** ^∗^	**0.678** ^∗∗^	-0.037	0.205	0.115	0.105
TG																**-0.497** ^∗∗^	-0.299	**0.516** ^∗∗^	**0.629** ^∗∗^	**0.566** ^∗∗^	**0.374** ^∗^
HDL-c																	**0.444** ^∗∗^	**-0.500** ^∗∗^	**-0.395** ^∗^	**-0.410** ^∗^	**-0.327** ^∗^
LDL-c																		**-0.432** ^∗∗^	-0.162	-0.238	-0.159
Glc																			**0.602** ^∗∗^	**0.742** ^∗∗^	0.257
Insulin																				**0.958** ^∗∗^	**0.394** ^∗^
HOMA																					**0.393** ^∗^

Spearman's correlation coefficients. Significant correlations are shown in bold (^∗^ *p* < 0.05, ^∗∗^ *p* < 0.01). BMI = body mass index; WC = waist circumference; HC = hip circumference; WHI = waist-hip index; W/Ht = waist-height ratio; SBP = systolic blood pressure; DBP = diastolic blood pressure; TC = total cholesterol; TG = triglycerides; HDL-c = high-density lipoprotein cholesterol; LDL-c = low-density lipoprotein cholesterol; Glc = glucose; HOMA-IR = homeostatic model assessment of insulin resistance.

## Data Availability

The values used to build the tables and graphs that support the findings of this study are available from the corresponding author upon request.

## Supplementary Materials

Supplementary Materials In order to address the detailed methodology, supplementary material is provided with a more extensive description of each laboratory study.

## References

[1] Dileepan K., Feldt M. M. (2013). Type 2 diabetes mellitus in children and adolescents. *Pediatrics in Review*.

[2] Huang P. L. (2009). A comprehensive definition for metabolic syndrome. *Disease Models & Mechanisms*.

[3] Hruby A., Hu F. B. (2015). The epidemiology of obesity: a big picture. *PharmacoEconomics*.

[4] Elder J. P. (2013). Mexico and the USA: the world’s leaders in the obesity epidemic. *Salud Pública de México*.

[5] Romero-Martínez M., Shamah-Levy T., Cuevas-Nasu L. (2017). Diseño metodológico de la Encuesta Nacional de Salud y Nutrición de Medio Camino 2016. *Salud Pública de México*.

[6] Bloomgarden Z. T. (2004). Type 2 diabetes in the young: the evolving epidemic. *Diabetes Care*.

[7] Baron P. F., Márquez E. (2010). Diabetes mellitus tipo 2 en niños y adolescentes. *Medicina Interna de México*.

[8] Gregor M. F., Hotamisligil G. S. (2011). Inflammatory mechanisms in obesity. *Annual Review of Immunology*.

[9] Kim C. S., Park H. S., Kawada T. (2006). Circulating levels of MCP-1 and IL-8 are elevated in human obese subjects and associated with obesity-related parameters. *International Journal of Obesity*.

[10] Azizian M., Mahdipour E., Mirhafez S. R. (2016). Cytokine profiles in overweight and obese subjects and normal weight individuals matched for age and gender. *Annals of Clinical Biochemistry*.

[11] Schmidt F. M., Weschenfelder J., Sander C. (2015). Inflammatory cytokines in general and central obesity and modulating effects of physical activity. *PLoS One*.

[12] Singer K., Lumeng C. N. (2017). The initiation of metabolic inflammation in childhood obesity. *The Journal of Clinical Investigation*.

[13] Chawla A., Nguyen K. D., Goh Y. P. S. (2011). Macrophage-mediated inflammation in metabolic disease. *Nature Reviews Immunology*.

[14] Ouchi N., Parker J. L., Lugus J. J., Walsh K. (2011). Adipokines in inflammation and metabolic disease. *Nature Reviews Immunology*.

[15] Wang X., Bao W., Liu J. (2012). Inflammatory markers and risk of type 2 diabetes: a systematic review and meta-analysis. *Diabetes Care*.

[16] Qiao Y. C., Shen J., He L. (2016). Changes of regulatory T cells and of proinflammatory and immunosuppressive cytokines in patients with type 2 diabetes mellitus: a systematic review and meta-analysis. *Journal of Diabetes Research*.

[17] Pedersen B. K., Febbraio M. A. (2012). Muscles, exercise and obesity: skeletal muscle as a secretory organ. *Nature Reviews Endocrinology*.

[18] Donath M. Y., Shoelson S. E. (2011). Type 2 diabetes as an inflammatory disease. *Nature Reviews Immunology*.

[19] Reinehr T., Roth C. L. (2018). Inflammation markers in type 2 diabetes and the metabolic syndrome in the pediatric population. *Current Diabetes Reports*.

[20] Reinehr T., Karges B., Meissner T. (2016). Inflammatory markers in obese adolescents with type 2 diabetes and their relationship to hepatokines and adipokines. *The Journal of Pediatrics*.

[21] Boström P., Wu J., Jedrychowski M. P. (2012). A PGC1-*α*-dependent myokine that drives brown-fat-like development of white fat and thermogenesis. *Nature*.

[22] Roca-Rivada A., Castelao C., Senin L. L. (2013). FNDC5/irisin is not only a myokine but also an adipokine. *PLoS One*.

[23] Perakakis N., Triantafyllou G. A., Fernández-Real J. M. (2017). Physiology and role of irisin in glucose homeostasis. *Nature Reviews Endocrinology*.

[24] Polyzos S. A., Anastasilakis A. D., Efstathiadou Z. A. (2018). Irisin in metabolic diseases. *Endocrine*.

[25] Mehrabian S., Taheri E., Karkhaneh M., Qorbani M., Hosseini S. (2015). Association of circulating irisin levels with normal weight obesity, glycemic and lipid profile. *Journal of Diabetes & Metabolic Disorders*.

[26] Park K. H., Zaichenko L., Brinkoetter M. (2013). Circulating irisin in relation to insulin resistance and the metabolic syndrome. *The Journal of Clinical Endocrinology & Metabolism*.

[27] Shoukry A., Shalaby S. M., el-Arabi Bdeer S., Mahmoud A. A., Mousa M. M., Khalifa A. (2016). Circulating serum irisin levels in obesity and type 2 diabetes mellitus. *IUBMB Life*.

[28] Crujeiras A. B., Pardo M., Arturo R. R. (2014). Longitudinal variation of circulating irisin after an energy restriction-induced weight loss and following weight regain in obese men and women. *American Journal of Human Biology*.

[29] Pardo M., Crujeiras A. B., Amil M. (2014). Association of irisin with fat mass, resting energy expenditure, and daily activity in conditions of extreme body mass index. *International Journal of Endocrinology*.

[30] Elizondo-Montemayor L., Mendoza-Lara G., Gutierrez-DelBosque G., Peschard-Franco M., Nieblas B., Garcia-Rivas G. (2018). Relationship of circulating irisin with body composition, physical activity, and cardiovascular and metabolic disorders in the pediatric population. *International Journal of Molecular Sciences*.

[31] Jang H. B., Kim H. J., Kang J. H., Park S. I., Park K. H., Lee H. J. (2017). Association of circulating irisin levels with metabolic and metabolite profiles of Korean adolescents. *Metabolism*.

[32] Binay Ç., Paketçi C., Güzel S., Samancı N. (2017). Serum irisin and oxytocin levels as predictors of metabolic parameters in obese children. *Journal of Clinical Research in Pediatric Endocrinology*.

[33] Palacios-González B., Vadillo-Ortega F., Polo-Oteyza E. (2015). Irisin levels before and after physical activity among school-age children with different BMI: a direct relation with leptin. *Obesity*.

[34] Elizondo-Montemayor L., Silva-Platas C., Torres-Quintanilla A. (2017). Association of irisin plasma levels with anthropometric parameters in children with underweight, normal weight, overweight, and obesity. *BioMed Research International*.

[35] Shim Y. S., Kang M. J., Yang S., Hwang I. T. (2018). Irisin is a biomarker for metabolic syndrome in prepubertal children. *Endocrine Journal*.

[36] Zhang C., Ding Z., Lv G., Li J., Zhou P., Zhang J. (2016). Lower irisin level in patients with type 2 diabetes mellitus: a case-control study and meta-analysis. *Journal of Diabetes*.

[37] Du X. L., Jiang W. X., Lv Z. T. (2016). Lower circulating irisin level in patients with diabetes mellitus: a systematic review and meta-analysis. *Hormone and Metabolic Research*.

[38] Levitt Katz L. E., Bacha F., Gidding S. S. (2018). Lipid profiles, inflammatory markers, and insulin therapy in youth with type 2 diabetes. *The Journal of Pediatrics*.

[39] American Diabetes Association (2018). 2. Classification and diagnosis of diabetes: standards of medical care in diabetes—2018. *Diabetes Care*.

[40] Centers for Disease Control and Prevention Defining childhood obesity. https://www.cdc.gov/obesity/childhood/defining.html.

[41] Centers for Disease Controls and Prevention, National Health and Nutrition Examination Survey (NHANES) (2007). *Anthropometry Procedures Manual*.

[42] Kurdiova T., Balaz M., Vician M. (2014). Effects of obesity, diabetes and exercise on Fndc5 gene expression and irisin release in human skeletal muscle and adipose tissue: in vivo and in vitro studies. *The Journal of Physiology*.

[43] Moreno-Navarrete J. M., Ortega F., Serrano M. (2013). Irisin is expressed and produced by human muscle and adipose tissue in association with obesity and insulin resistance. *The Journal of Clinical Endocrinology & Metabolism*.

[44] Liu J.-J., Wong M. D. S., Toy W. C. (2013). Lower circulating irisin is associated with type 2 diabetes mellitus. *Journal of Diabetes and its Complications*.

[45] Choi Y.-K., Kim M. K., Bae K. H. (2013). Serum irisin levels in new-onset type 2 diabetes. *Diabetes Research and Clinical Practice*.

[46] Huh J. Y., Dincer F., Mesfum E., Mantzoros C. S. (2014). Irisin stimulates muscle growth-related genes and regulates adipocyte differentiation and metabolism in humans. *International Journal of Obesity*.

[47] Xiong X. Q., Chen D., Sun H. J. (2015). FNDC5 overexpression and irisin ameliorate glucose/lipid metabolic derangements and enhance lipolysis in obesity. *Biochimica et Biophysica Acta (BBA) - Molecular Basis of Disease*.

[48] Huh J. Y., Mougios V., Kabasakalis A. (2014). Exercise-induced irisin secretion is independent of age or fitness level and increased irisin may directly modulate muscle metabolism through AMPK activation. *The Journal of Clinical Endocrinology & Metabolism*.

[49] Park M. J., Kim D. I., Choi J. H., Heo Y. R., Park S. H. (2015). New role of irisin in hepatocytes: the protective effect of hepatic steatosis in vitro. *Cellular Signalling*.

[50] Batirel S., Bozaykut P., Mutlu Altundag E., Kartal Ozer N., Mantzoros C. S. (2014). OP2-4 - the effect of irisin on antioxidant system in liver. *Free Radical Biology & Medicine*.

[51] Liu T. Y., Shi C. X., Gao R. (2015). Irisin inhibits hepatic gluconeogenesis and increases glycogen synthesis via the PI3K/Akt pathway in type 2 diabetic mice and hepatocytes. *Clinical Science*.

[52] Mootha V. K., Lindgren C. M., Eriksson K. F. (2003). PGC-1*α*-responsive genes involved in oxidative phosphorylation are coordinately downregulated in human diabetes. *Nature Genetics*.

[53] Delarue J., Magnan C. (2007). Free fatty acids and insulin resistance. *Current Opinion in Clinical Nutrition and Metabolic Care*.

[54] Gamas L., Matafome P., Seiça R. (2015). Irisin and myonectin regulation in the insulin resistant muscle: implications to adipose tissue: muscle crosstalk. *Journal Diabetes Research*.

[55] Riwanto M., Landmesser U. (2013). High density lipoproteins and endothelial functions: mechanistic insights and alterations in cardiovascular disease. *Journal of Lipid Research*.

[56] Shah A. S., Urbina E. M. (2017). Vascular and endothelial function in youth with type 2 diabetes mellitus. *Current Diabetes Reports*.

[57] De Meneck F., Victorino de Souza L., Oliveira V., do Franco M. C. (2018). High irisin levels in overweight/obese children and its positive correlation with metabolic profile, blood pressure, and endothelial progenitor cells. *Nutrition, Metabolism, and Cardiovascular Diseases*.

[58] Stengel A., Hofmann T., Goebel-Stengel M., Elbelt U., Kobelt P., Klapp B. F. (2013). Circulating levels of irisin in patients with anorexia nervosa and different stages of obesity – correlation with body mass index. *Peptides*.

[59] Löffler D., Müller U., Scheuermann K. (2015). Serum irisin levels are regulated by acute strenuous exercise. *The Journal of Clinical Endocrinology & Metabolism*.

[60] Huh J. Y., Panagiotou G., Mougios V. (2012). FNDC5 and irisin in humans: I. Predictors of circulating concentrations in serum and plasma and II. mRNA expression and circulating concentrations in response to weight loss and exercise. *Metabolism*.

[61] Çatlı G., Küme T., Tuhan H. Ü. (2016). Relation of serum irisin level with metabolic and antropometric parameters in obese children. *Journal of Diabetes and its Complications*.

[62] Nigro E., Scudiero O., Ludovica Monaco M. (2017). Adiponectin profile and irisin expression in Italian obese children: association with insulin-resistance. *Cytokine*.

[63] Blüher S., Panagiotou G., Petroff D. (2014). Effects of a 1-year exercise and lifestyle intervention on irisin, adipokines, and inflammatory markers in obese children. *Obesity*.

[64] Reilly S. M., Saltiel A. R. (2017). Adapting to obesity with adipose tissue inflammation. *Nature Reviews Endocrinology*.

[65] Sell H., Habich C., Eckel J. (2012). Adaptive immunity in obesity and insulin resistance. *Nature Reviews Endocrinology*.

[66] Calle M. C., Fernandez M. L. (2012). Inflammation and type 2 diabetes. *Diabetes & Metabolism*.

[67] Lackey D. E., Olefsky J. M. (2016). Regulation of metabolism by the innate immune system. *Nature Reviews Endocrinology*.

[68] Askari H., Rajani S. F., Poorebrahim M., Haghi-Aminjan H., Raeis-Abdollahi E., Abdollahi M. (2018). A glance at the therapeutic potential of irisin against diseases involving inflammation, oxidative stress, and apoptosis: an introductory review. *Pharmacological Research*.

[69] Aguiar F. J. B., Ferreira-Júnior M., Sales M. M. (2013). C-reactive protein: clinical applications and proposals for a rational use. *Revista da Associação Médica Brasileira (English Edition)*.

[70] Mazur-Bialy A. I., Pocheć E., Zarawski M. (2017). Anti-inflammatory properties of irisin, mediator of physical activity, are connected with TLR4/MyD88 signaling pathway activation. *International Journal of Molecular Sciences*.

[71] Mazur-Bialy A. I., Bilski J., Pochec E., Brzozowski T. (2017). New insight into the direct anti-inflammatory activity of a myokine irisin against proinflammatory activation of adipocytes. Implication for exercise in obesity. *Journal of Physiology and Pharmacology*.

[72] Xiong X.-Q., Geng Z., Zhou B. (2018). FNDC5 attenuates adipose tissue inflammation and insulin resistance via AMPK-mediated macrophage polarization in obesity. *Metabolism*.

[73] Dong J., Dong Y., Dong Y., Chen F., Mitch W. E., Zhang L. (2016). Inhibition of myostatin in mice improves insulin sensitivity via irisin-mediated cross talk between muscle and adipose tissues. *International Journal of Obesity*.

[74] Matsuo Y., Gleitsmann K., Mangner N. (2015). Fibronectin type III domain containing 5 expression in skeletal muscle in chronic heart failure-relevance of inflammatory cytokines. *Journal of Cachexia, Sarcopenia and Muscle*.

[75] Viitasalo A., Atalay M., Pihlajamäki J. (2015). The 148 M allele of the *PNPLA3* is associated with plasma irisin levels in a population sample of Caucasian children: the PANIC study. *Metabolism*.

[76] Sarac F., Buyukbese Sarsu S., Yeniocak S. (2018). The diagnostic value of irisin in pediatric patients with acute abdominal pain. *Emergency Medicine International*.

[77] Sproston N. R., Ashworth J. J. (2018). Role of C-reactive protein at sites of inflammation and infection. *Frontiers in Immunology*.

[78] Mazur-Bialy A. I. (2017). Irisin acts as a regulator of macrophages host defense. *Life Sciences*.

[79] Abedini A., Schmidt A. M. (2013). Mechanisms of islet amyloidosis toxicity in type 2 diabetes. *FEBS Letters*.

[80] Yang M., Chen P., Jin H. (2014). Circulating levels of irisin in middle-aged first-degree relatives of type 2 diabetes mellitus — correlation with pancreatic *β*-cell function. *Diabetology & Metabolic Syndrome*.

