# Supplementary material for: Association between Irisin, hs-CRP, and Metabolic Status in Children and Adolescents with Type 2 Diabetes Mellitus
Source: Mediators Inflamm. 2019 Mar 20;2019:6737318. doi: 10.1155/2019/6737318 (PMC6446111; doi:10.1155/2019/6737318)
Supplement: Supplementary Materials — In order to address the detailed methodology, supplementary material is provided with a more extensive description of each laboratory study. [file 6737318.f1.docx]

**Supplementary Material**

**Laboratory studies**

Blood samples were taken from each subject by peripheral venipuncture after an overnight 12-hour fast. Samples were then centrifuged to obtain plasma and serum and were then frozen at −80°C for further processing.

Fasting serum glucose levels were measured by the Hexokinase (HK)/Glucose-6-Phosphate dehydrogenase (G-6-PDH) method, running the Glucose 3L82 (304772/R02; DENKA SEIKEN CO., LTD. Tokyo Japan) reagent kit on the Architects *c*Systems™. HK/ G-6-PDH method’s principle consists of nicotinamide adenine dinucleotide reduced (NADH) [product of the concurrent reduction of nicotinamide adenine, as G-6-PDH oxidizes G-6-P, which is obtained from the phosphorylation of glucose by hexokinase] absorbing light at 340 nm and detecting it as an increased absorbance by spectrophotometry. For each micromole of glucose consumed, one micromole of NADH is produced.

Serum insulin concentrations were obtained by chemiluminescent microparticle immunoassay, using the ARCHITECT Insulin Reagent kit 8K41-27 (G6-2892/R03); Abbot Laboratories Diagnostic Division, IL USA). The methodology consists of combining anti-insulin coated microparticles from the sample and anti-insulin acridinium labeled conjugate; insulin from the sample binds to the insulin coated microparticles and anti-insulin acridinium labeled conjugate. As pre-trigger and trigger solutions are added to the mixed reaction after washing, the culminating chemiluminescent reaction is measured as Relative Light Units (RLUs); A relationship between the concentration of insulin in the sample and RLUs is then detected by the ARCHITECT immunoassay optical system.

Total Cholesterol was measured running the Cholesterol Reagent kit 7D62 (304796/R02; Abbot Laboratories Diagnostic Division, IL USA) on the Architect *c*Systems™. The method consists of enzymatically hydrolyzation of cholesterol esters to cholesterol and free fatty acids by cholesterol esterase. An oxidation reaction is then performed by cholesterol oxidase from free cholesterol to cholest-4-ene-3-one and hydrogen peroxide. As hydrogen peroxide combines with hydroxybenzoic acid and 4-minoantypirine, a quinoneimine dye is formed and quantitated at 500 nm.

High-density lipoprotein cholesterol concentrations were measured by the accelerator selective detergent method using the Ultra HDL 3K33-21 assay (306571/R03; Abbot Laboratories Diagnostic Division, IL USA). The method consists of accelerating the reaction of non-HDL unsterified cholesterol with cholesterol oxidase and using a detergent to dissolve HDL cholesterol. Two reagents are used; the first one subjects non-HDL unesterifed to an enzymatic reaction, generating peroxide, which is consumed by a reaction with DSmbt, producing a colorless product. The second reagent is conformed by a detergent, a cholesterol esterase and a chromogenic coupler, which develops color to quantify HDL-C.

Quantitation of triglycerides in plasma was obtained through the glycerol-phosphate-oxidase reaction, running the Triglyceride 7D74-20 (30-3140/R3; Abbot Laboratories Diagnostic Division, IL USA) reagent kit on the Architect *c*Systems™ and the AEROSET system. The method consists of an enzymatic reaction carried out by lipase, in which triglycerides are hydrolyzed to free fatty acids and glycerol. Glycerol kinase phosphorylates glycerol with adenosine triphosphate (ATP) to obtain glycerol-3-phosphate and adenosine diphosphate (ADP). An oxidation reaction from glycerol-3- phosphate to dihydroxyacetone phosphate is carried out by phosphate oxidize, producing hydrogen peroxide, which reacts with 4-chlorophenol and 4-aminoantipyrine to produce a red dye. The red colored dye’s absorbance is proportional to the concentration of triglycerides.

High-Sensitivity C-reactive Protein levels were determined by quantitative immunoturbidimetric methodology, using the CRP Vario 6k26-30 and 6k26-41 kits (306731/R04; Abbot Laboratories Diagnostic Division, IL USA) on the Architect *c*Systems™. CRP Vario is a latex immunoassay; when CRP reacts with a CRP antibody adsorbed by the latex particles, an agglutination reaction results and is detected by an absorbance change, which is proportional to the CRP concentrations of the sample.

Irisin was measured by sandwich enzyme-linked immunosorbent assay (ELISA) method with an irisin (human) ELISA kit (SK00170-08) following the manufacturer’s instructions (Avisera Bioscience Inc, Santa Clara, California, USA). The sensitivity of this assay is 0.1 ng/mL, its standard curve linear range is 0.8-51.2 ng/mL and intra- and inter-assay variation are 4-6% and 8-10% respectively. The method consists of a specific monoclonal antibody pre-coated to a plate, which can bind to irisin in the standard and samples. The unbound substances are washed off the plate and a monoclonal antibody against human irisin is aggregated to the wells. The plate is washed again and a Treptavidin-HRP Conjugate is added. After a third wash, TMB, a substrate solution is aggregated to the wells and color is developed, which is proportional to the concentration of human irisin in the samples or standard solution. A standard curve was generated to read the sample values.

For cytokine measurement, Human TNF-α (430306) and Human IL-6 ELISA MAX (430503) sets, both from Biolegend (San Diego, CA, USA). Sensitivity for the assay is 2 pg/mL. The Human TNF-α and Human IL-6 ELISA MAX assays consist of sandwich Enzime-Linked Immunosorbent assays, which were performed following the manufacturer’s instructions (BioLegend, San Diego, CA, USA). The method consists of adding a specific human TNF-α/IL-6 mouse monoclonal antibody to a precoated plate. Standard and samples are then added to the wells and the TNF-α/IL-6 binds to the capture antibody. A detection anti-TNF-α/ anti-IL-6 antibody is aggregated, and a sandwich conformed by antibody, antigen and antibody is formed. Thereafter, Avidin-horseradish peroxidase and TMB substrate solutions are added, and a blue color proportional to the TNF-α/IL-6 sample’s concentration is produced. Ultimately, the Stop Solution transforms the color from blue to yellow, and the absorbance is read at 450 nm with a microplate reader.
